# A Review of Posttraumatic Bowel Injuries in Ibadan

**DOI:** 10.5402/2011/478042

**Published:** 2011-08-17

**Authors:** A. E. Dongo, E. B. Kesieme, D. O. Irabor, J. K. Ladipo

**Affiliations:** ^1^Department of Surgery, Irrua Specialist Teaching Hospital, PMB 8, Irrua, Edo State, Nigeria; ^2^Department of Surgery, University College Hospital, PMB 5017, Ibadan, Oyo State, Nigeria

## Abstract

*Background*. Bowel injuries are a leading cause of morbidity and mortality following trauma. Evaluating patients who sustained abdominal trauma with bowel injury may pose a significant diagnostic challenge to the surgeon. Prompt recognition and timely intervention is necessary to improve outcome. *Aim*. This study was undertaken to evaluate treatment and outcome of patients with bowel trauma. *Methods*. A 5-year retrospective study of all patients presenting with abdominal trauma requiring surgical intervention seen in the UCH Ibadan, Nigeria was undertaken. *Results*. There were 71 patients (59 males and 12 females). The majority of cases (70%) occurred between the 3rd and 5th decades of life. Some 37 patients (52%) sustained blunt abdominal injury, while 34 patients (48%) sustained penetrating abdominal injury. There were 27 patients with bowel injuries (38%). Isolated bowel injuries occurred in 19 patients (27%). The most common surgical operation performed was simple closure. There were 3 deaths in patients with bowel injuries. *Conclusion*. Most cases of bowel injury can be managed by simple closure, a technique that is not so technically demanding for surgeons in less-developed countries. This study has also incidentally identified a “rule of six” for patients with bowel injuries and abdominal trauma.

## 1. Introduction

Posttraumatic bowel injuries have been recognized since ancient times. But it was not until 1834 that a French Surgeon, M. L. Baudens, performed the first exploratory laparotomy for trauma [1, 2]. Since then, management has gone full cycle from exploration for all cases of penetrating trauma to the present maxim that “not everybody with a hole in the abdomen needs exploration” [3]. 

Bowel injuries may result from either a blunt or penetrating abdominal injury. Blunt trauma cause injuries by either compression or by deceleration. Compression forces can result in transient increase in intraluminal pressure resulting in rupture especially of the small bowel. Following blunt abdominal trauma, deceleration injuries cause small bowel injuries typically to occur where mobile and fixed segments are attached and are prone to shear force injury, that is, the proximal jejunum near the ligament of Treitz or at the distal ileum near the ileocaecal junction [4]. Munns et al. showed that following blunt trauma, the most common small bowel injury was “blowout” perforation on the antimesenteric border of the bowel (55.5%), while the most common colonic injury was a serosal tearbruise (62.2%) [5].

Penetrating abdominal trauma may result from firearm, knives, and broken glass pieces. 80% of penetrating injuries are due to firearm, and 20% are due to stab wounds [6]. These injuries are common in war victims, and they cause multiple organ injuries. The colon and small intestine were the most commonly injured organs and had the most postoperative complications [7]. Gunshot wounds and shotgun blasts are by far more destructive and have a higher degree of morbidity and mortality than stab wounds. Gunshot wounds and other projectiles have a higher degree of energy and produce fragmentation as a direct effect and cavitation, resulting in greater organ injury.

Bowel injuries may occur alone or in association with other injuries involving the mesentery, liver, spleen, kidney, and pancreas

Generally, diagnostic modalities in abdominal injuries include focused abdominal sonography for trauma (FAST), computerized tomographic scan, and diagnostic peritoneal lavage. Where these facilities are unavailable or unaffordable as in developing countries, the surgeon has no option than to perform an early exploration.

Adherence to standard surgical protocols, proper evaluation, and management are factors which may lessen complications of bowel injuries associated with abdominal trauma. Delayed diagnosis of bowel injury will result in peritonitis and sepsis.

We undertook this study to evaluate treatment and outcome of patients with bowel trauma in our locality. We believe that the result will help us in understanding the peculiarity of bowel trauma in a background of a developing nation and will help us to draw appropriate conclusions.

## 2. Methods

We undertook a retrospective study of all patients presenting with abdominal trauma and requiring surgical intervention seen in the University College Hospital Ibadan, Nigeria, between September 1st 1999 and August 31st 2005.

University college Hospital, Ibadan, is the premier teaching hospital in Nigeria. It is a 500-bedded hospital situated in Ibadan, a town with a population of 1,338,659 according to the 2006 census. It serves as a referral center for other general hospitals in Oyo State and teaching hospitals in South-Western Nigeria.

Patients presenting with abdominal trauma are admitted through the Accident and Emergency Unit and are first reviewed and resuscitated by the General Surgical Unit on call.

Data were obtained from the operation records and the surgical units' admission diaries. Those with small bowel injuries were identified and divided into isolated or nonisolated groups. The nonisolated group comprised those with bowel injuries and other intra-abdominal injuries. 

Demographics obtained include age, sex, surgical procedure, surgical outcome (deaths and other intraabdominal complications), and length of hospital stay. 

The data collected were analyzed using descriptive analysis and percentages.

## 3. Results

Of the 71 patients who sustained abdominal trauma requiring operative intervention, 27 had bowel injuries. This constituted 38% of all cases of trauma seen. There were 59 males and 12 females, giving a male to female ratio of 6 : 1. The age range was between 13 and 65 years.

**Figure 1:**
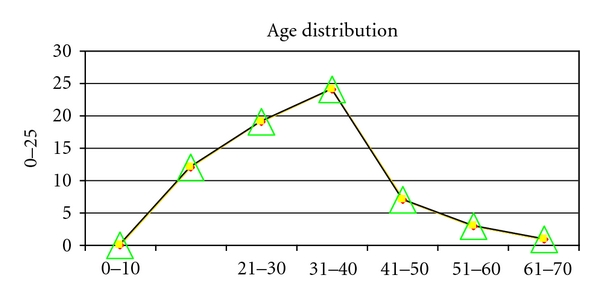
Age distribution.

The majority of cases occurred between the 3rd and 5th decades of life (Figure 1) with about 70% of patients involved within this age bracket.

**Figure 2:**
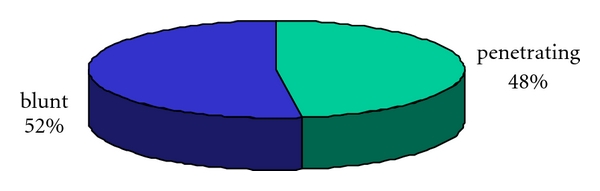
Distribution of blunt and penetrating injuries.

The ratio of blunt to penetrating trauma was almost equal 37 patients (52%) to 34 patients (48%), respectively (Figure 2). Among patients with penetrating injury the commonest cause was the gunshot injury, with 29 (40%) cases and stab injury with 5 (7%) cases.

**Figure 3:**
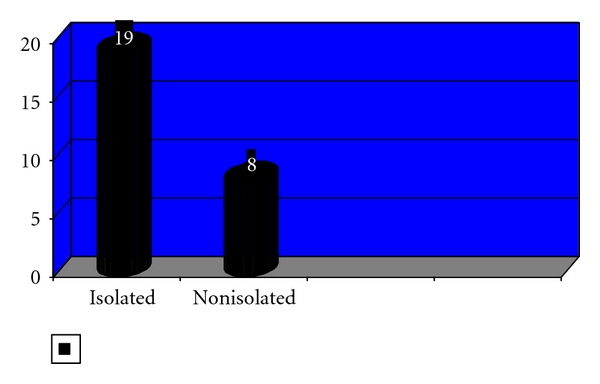
Distribution of isolated versus nonisolated injuries.

The small bowel was the only organ injured in 19 (26%) cases, and it was associated with other intra-abdominal organs in 8 (10%) patients (Figure 3).

The mean duration of hospital stay was 13.11 + 4.3 days. Five patients stayed beyond 3 weeks on admission. The majority of the cases were discharged within 2 weeks. Three deaths were recorded. One with isolated bowel injury presenting after 3 days, while 2 had colonic injuries with other intra-abdominal injuries. Eleven patients had simple closure and lavage, and 6 patients had bowel resection and anastomosis. Five patients had right hemicolectomy, and another 5 colostomies fashioned for left-sided colonic injuries. None of the patient has been readmitted for either short bowel syndrome or intestinal adhesions. From our results, we hypothesized “a rule of 6” for abdominal trauma and bowel injuries.

1/6 of patients with abdominal trauma are females.
1/6 of patients who sustained penetrating wounds are be due to stab wounds.
2/6 of patients will sustain bowel trauma after abdominal trauma.
3/6 of patients would have either penetrating trauma or blunt trauma.
4/6 of patients would be within the 3rd and 5th decades of life.
5/6 of patients would be discharged within 2 weeks of hospital stay.

## 4. Discussion

The small and large bowel are commonly affected in penetrating trauma [8, 9]. However, they are less frequently affected than solid organs like the spleen and the liver in blunt trauma [9]. Blunt abdominal trauma is known to be commonest after vehicular accident [10, 11]. Falls play a minor but significant role. Penetrating trauma is on the increase in Africa with strife especially in conflict zone, as well as in civilian life from armed robbery attacks [12], while in the Western world, the incidence of penetrating abdominal trauma appears to be on the decline [13]. In our study, we found that majority of our patients who sustained abdominal trauma were males. This agrees with other studies in which males consistently outnumber females [10, 12]. The majority (68.82%) were within the young and active age group, that is, the 3rd and 5th decades of life. This also agrees with the result of Alli in a similar study in Maiduguri, Nigeria [14].

Bowel Injury was identified in 27 of our patients (38%) with abdominal trauma. This agrees with a ratio of 34% identified by Hackman et al. [15]. The ratio of patients with blunt to abdominal trauma was almost equal 1.1 : 1. This contrasts with generally accepted ratio of 2 : 1 [15] quoted in civilian life, a trend that is found to be increasing in favour of blunt abdominal trauma [16, 17]. Whether this is due to a reduction in the number of blunt trauma or an increase in penetrating trauma in our study would need further study to be evaluated. Among patients with penetrating trauma, gunshot injury was the commonest cause in 29 patients. Stab wounds were relatively rare occurring in only 5 patients, giving a ratio of stab wound to gunshot wound of 1 : 6.

The majority of patient (70%) with bowel trauma had isolated bowel injuries. Most patients with isolated bowel trauma injury usually make uncomplicated recovery [18]. The only death recorded in this subset of patients was in a patient presenting 3 days after bowel injury. It has been clearly demonstrated that delay in presentation even as little as 8 hours adversely affects outcome following small bowel injury [19].

The commonest procedure carried out was simple closure of perforated small bowel injuries. This is a well established procedure with minimal complications [20]. Large bowel injuries were treated with either a right hemicolectomy or a colostomy for left-sided colonic injuries. There was no case of simple repair of colonic injury despite the present recommendation of simple repair for nondestructive colonic injuries [21–23] Furthermore, colostomies are known to be more costly with poorer life quality when compared with simple repair [24].

Most patients made uneventful recovery with the majority being discharged with 2 weeks. Only 3 patients remained longer than 3 weeks on admission.

There were 3 deaths recorded in all. Two of these deaths occurred in patients with nonisolated injuries. All the 3 deaths occurred within 1 week of admission, 2 from overwhelming sepsis, and one from multiple organ failure.

## 5. Conclusion

Injury to the bowel requires adequate evaluation. Simple closure can be done for the majority of these injuries. A rule of 6 can be described for bowel injuries in abdominal trauma.
